# Nicotinic Acid Receptor GPR109A Is Down-Regulated in Human Macrophage-Derived Foam Cells

**DOI:** 10.1371/journal.pone.0062934

**Published:** 2013-05-02

**Authors:** Joshua T. Chai, Janet E. Digby, Neil Ruparelia, Andrew Jefferson, Ashok Handa, Robin P. Choudhury

**Affiliations:** 1 Division of Cardiovascular Medicine, University of Oxford, Oxford, United Kingdom; 2 Nuffield Department of Surgical Sciences, University of Oxford, Oxford, United Kingdom; University of Padova, Italy

## Abstract

Nicotinic acid (NA) regresses atherosclerosis in human imaging studies and reduces atherosclerosis in mice, mediated by myeloid cells, independent of lipoproteins. Since GPR109A is expressed by human monocytes, we hypothesized that NA may drive cholesterol efflux from foam cells. In THP-1 cells NA suppressed LPS-induced mRNA transcription of MCP-1 by 76.6±12.2% (P<0.01) and TNFα by 56.1±11.5% (P<0.01), yet restored LPS-induced suppression of PPARγ transcription by 536.5±46.4% (P<0.001) and its downstream effector CD36 by 116.8±19.8% (P<0.01). Whilst direct PPARγ-agonism promoted cholesterol efflux from THP-1 derived foam cells by 37.7±3.1% (P<0.01) and stimulated transcription of LXRα by 87.9±9.5% (P<0.001) and ABCG1 by 101.2±15.5% (P<0.01), NA showed no effect in foam cells on either cholesterol efflux or key RCT genes transcription. Upon foam cell induction, NA lost its effect on PPARγ and cAMP pathways, since its receptor, GPR109A, was down-regulated by foam cell transformation. This observation was confirmed in explanted human carotid plaques. In conclusion, despite NA’s anti-inflammatory effect on human macrophages, it has no effect on foam cells in reverse cholesterol transport; due to GPR109A down-regulation.

## Introduction

For over fifty years, nicotinic acid (NA) has been known to exert favourable effects on plasma lipoproteins [1]. It reduces atherogenic lipoproteins LDL-c, VLDL-c, and Lp(a), and is the most efficacious drug currently available to raise plasma HDL-c (by up to 30%) [2]. The Coronary Drug Project [3] has shown benefits of NA in reducing cardiovascular events and mortality in patients with coronary heart disease, a finding supported by 2 recent meta-analyses [4], [5]. Furthermore, numerous trials have shown that NA reduces atherosclerosis, estimated from coronary angiography [6], [7]; carotid ultrasound [8]–[11] and MRI [12]. However, one recent outcome trial was abandoned due to lack of efficacy [13]. A larger Phase III trial of a niacin/laropiprant combination also did not reach its primary end point [14].

In addition to the recognized effects on plasma lipoproteins, it has emerged that NA has a variety of additional actions that are of potential relevance to processes of atherosclerosis progression and regression. In adipocytes [15] and certain non-foam cell ‘basal-state’ mononuclear cell lines [16], [17], NA activates the reverse cholesterol transport apparatus, namely PPARγ, LXRα, and the ABC transporter proteins, which are known to be responsible for *in vitro* cholesterol unloading [18] and, in turn, are directly implicated in the regression of experimental atherosclerosis [19], [20]. These observations raise the possibility that effects of NA on atherosclerotic plaque regression and cardiovascular risk reduction may, in part, be due to direct effects on foam cell cholesterol unloading.

GPR109A (also known as hydroxy-carboxylic acid 2 receptor or HM74a) which is the receptor for nicotinic acid, has been shown to mediate an expanding repertoire of potentially therapeutic actions [21], [22]. This receptor belongs to a family of Gi-protein-coupled cell surface receptors that are expressed in adipocytes and immune cells in both human and rodent species [21]. We have recently reported potent anti-inflammatory effects of NA in both human adipocytes [23] and monocytes [24], which are mediated via GPR109A-dependent mechanisms. The relevance of these observations has been enhanced by a recent study in mice, which has shown that NA reduces progression of atherosclerosis, via GPR109A on myeloid cells, without affecting plasma lipoproteins [25].

The observations that, NA (i) plays a role in atherosclerosis regression in humans and (ii) has the capacity to act directly on monocyte/macrophage function by up-regulating proteins that are involved in cellular cholesterol efflux, raise the important possibility that NA may exert effects, via its receptor GPR109A, in foam cells to induce cholesterol efflux, leading to plaque regression. Accordingly, we sought to test the effects of NA on human macrophage-derived foam cells and explore its cellular mechanisms of action in the context of lipid handling and atherogenesis.

## Methods

### Cell Culture and Treatment

THP-1 cells were purchased from American Type Culture Collection (ATCC, Teddington, UK) and maintained in RPMI 1640 medium (Sigma-Aldrich, Poole, UK) until treatment, at a density between 4–8×10^5^ cells/mL, supplemented with fetal bovine serum (10%, Invitrogen, Paisley, UK) and 2-mercaptoethanol (0.05 mM, Sigma-Aldrich, Poole, UK), in a humidified atmosphere of 95% air/5% CO_2_ at 37°C. Cells were treated with nicotinic acid (Sigma-Aldrich, Poole, UK) at 1×10^−3^ to 10^−6^ M or PPARγ agonist GW1929 (2×10^−6^ M, Tocris Bioscience, Bristol, UK) versus vehicle-only controls.

### *In vitro* Cholesterol Loading and Efflux

THP-1 monocytic cells were seeded in 96-well or 6-well plates at 1×10^6^ cells/mL density and allowed to differentiate for 48 hours into basal macrophages, in the presence of 50 ng/mL phorbol myristate acetate (PMA). For cholesterol loading and foam cell induction, basal macrophages were washed once with warm PBS and serum-starved for 12 hours, followed by incubation with acetylated (ac−) LDL (50 µg/mL, Biomedical Technologies, Inc, USA) for 48 hours (Figure S1 shows cell treatment regime in diagrammatic details). Foam cell formation was confirmed by Oil-red-O (Sigma-Aldrich, Poole, UK) staining in sample wells. Cholesterol efflux was assessed using HDL3 (50 µg/mL, Meridian Life Science, USA) and apolipoprotein (Apo) AI (10 µg/mL, Sigma-Aldrich, Poole, UK) as cholesterol acceptors and a non-radioactive enzymatic method (Amplex Red Cholesterol Assay kit, Invitrogen, Paisley, UK) modified from Robinet, *et al.* [26]. In brief, after the indicated treatment period, cell culture supernatants were collected and stored for later analysis; while foam cells were washed in cold PBS twice and cellular cholesterol extracted by incubation with hexane:isopropanol (3∶2) solvent mixture with gentle agitation for 30 minutes. This solvent mixture was then transferred to 1.5 mL Eppendorf microtubes for suction evaporation with the dried cholesterol residue reconstituted in an enzyme-compatible solvent system isopropanol:Nonidat P40 (9∶1, Roche, Burgess Hill, UK), supplemented with 20 U/mL of bovine catalase (Sigma-Aldrich, Poole, UK) to quench intrinsic peroxide activity. Extracted cellular and supernatant cholesterol content was then determined using Amplex Red Cholesterol Assay kit (Invitrogen, Paisley, UK) as per manufacturer’s instruction.

### mRNA Extraction and Quantitative Real-time RT-PCR

Total RNA was prepared using Qiagen® (Crawley, UK) RNEasy mini columns and 1 µg was reverse transcribed using a QuantiTect® Reverse Transcription Kit with Oligo dTs and random hexamers as primers. Real-time PCR was carried out with 1 µL of cDNA in a 10 µL reaction mix using custom primers with SYBR Green PCR master mix, or with Taqman™ Gene Expression assays (Applied Biosystems, Warrington, UK). Quantification was performed by the -ΔΔCT method [27], normalized to the housekeeping gene cyclophilin. Custom designed primers (5′- to 3′-) for PPARγ forward GATTTCACTATGGAGTTCATGCTTGT, reverse CATCTGTCATAGATAAG-CTTCAATCTGA; NR1H3 (for LXRα) forward AAGCCCTGCATGCCTACGT, reverse GCATCCGTGGGAACATCAG; ABCA1 forward GACAAATAAAAT-CAAGGATGGGTACTG, reverse AGACGTACCGCATGTCCTCAA; ABCG1 forward TGTCGGCACATCTGAAGCTT, reverse TGTCAGTATCTCCTTGA-CCATTTCC; GPR109A forward GCGTTGGGACTGGAAGTTTG, reverse GCGGTTCATAGCCAACATGA. Taqman™ Gene Expression assay primers from Applied Biosystems, Warrington, UK, were used for TNFα (Hs00174128_m1), MCP-1 (Hs00234140_m1), and CD36 (Hs00169627_m1).

### PPAR Transcription Factor Binding Assay

After treatment, THP-1 basal macrophages and foam cells were washed and total nuclear protein lysates prepared using Nuclear Extraction Kit (Millipore, Watford, UK). Nuclear protein contents were quantified by BCA assay (Thermo Scientific, Hemel Hempstead, UK) and 3 µg of total nuclear protein per sample was used to evaluate PPAR transcription factor binding activity using PPARα, δ, γ Complete Transcription Factor Assay Kit (Cayman Chemical, Michigan, USA) as per manufacturer’s instruction.

### Cyclic-AMP Assay

To determine the optimal treatment time period, basal macrophages were first incubated with NA at 1×10^−4^ M with duration ranging from 0 to 24 hours. Cellular cAMP concentration was determined using the Parameter™ Cyclic AMP ELISA kit (R & D Systems, Abingdon, UK). To evaluate G_i/o_-protein involvement, cells were pre-incubated with pertussis toxin (100 ng/mL, Calbiochem, Merck, Nottingham, UK) versus vehicle for 18 hours, prior to treatment with NA for the optimal time period determined previously.

### Western Blot

Cells in culture dish were harvested and lysed with CelLytic™ cell lysis reagent (Sigma-Aldrich, Poole, UK) with Complete™ protease inhibitor cocktail (Roche, Burgess Hill, UK) before total cell lysate protein quantification by BCA assay (Thermo Scientific, Hemel Hempstead, UK). 12 µg of total lysate protein were denatured and loaded in NuPage gel (Invitrogen, Paisley, UK) for electrophoresis. Protein bands were transferred to membrane using iBlot dry transfer system (Invitrogen, Paisley, UK), and probed with rabbit anti-human GPR109A (1∶1000, Imgenex, USA) primary antibody in PBS-Tween +5% BSA at 4°C overnight. After three washes, the membrane was incubated with anti-rabbit IgG HRP secondary antibody (1∶2000, GE Healthcare, Amersham, UK) at room temperature for 1 hour. Antibody complexes were visualised using ECL advanced detection kit (GE Healthcare, Amersham, UK). Loading control was performed using antibody against β-tubulin.

### *Ex-vivo* Human Carotid Atherosclerotic Plaque

Carotid atherosclerotic plaque samples were obtained from patients undergoing carotid endarterectomy in routine clinical care at Oxford University Hospital, with full written informed consent. Ethics approval was obtained from National Research Ethics Services (NRES) and local R&D committee prior to the beginning of the study and conformed to the Declaration of Helsinki. At the time of carotid endarterectomy, plaque samples were freshly divided. A small part of each plaque was formalin fixed, processed, and paraffin embedded (FFPE) for Masson’s trichrome staining for plaque morphology; while the remaining segments of the plaque were snap-frozen in OCT. Paraffin sections (5 µm) and frozen sections (10 µm) were obtained for histological and immunohistochemistry staining. Oil-red-O (Sigma-Aldrich, Poole, UK) staining was performed in selected co-staining experiments.

### Fluorescence Immunocytochemistry

Cultured cells or air-dried cryosections were fixed in ice-cold acetone for 10 minutes, washed with PBS, and blocked with serum-free protein blocking solution (Dako, Cambridge, UK) for 2 hours in room temperature. They were then incubated with mouse anti-human CD68 antibodies (1∶300, Dako, Cambridge, UK), rabbit anti-human GPR109A antibodies (1∶200, Imgenex, USA), and goat anti-human adipophilin antibodies (1∶100, Santa Cruz Biotechnology, Santa Cruz, CA, USA) at 4°C overnight. After washing, cells/cryosections were incubated with donkey anti-mouse IgG Alexa Fluor 647, (Invitrogen, Paisley, UK), donkey anti-rabbit IgG Alexa Fluor 594 (Invitrogen, Paisley, UK), and donkey anti-goat IgG Alexa Fluor 488 (Invitrogen, Paisley, UK) secondary antibodies at room temperature for 1 hour. After DAPI/TO-PRO3 nuclear counterstain, images were taken using a Leica DM2500 microscope with QImaging MicroPublisher 5.0 RTV image sensor, and a Leica SP-5 laser-scanning confocal microscope. To ensure adipophilin antibody binding specificity, competitive peptide blocking experiment was performed with the peptide from Santa Cruz Biotechnology used to raise its anti-adipophilin antibody. In lipid co-staining experiments, the immediately adjacent cryosection was fixed in 4% PFA for 1 hour, and stained with 36% solution of Oil-red-O (ORO) in triethyl phosphate (Sigma-Aldrich, Poole, UK) for 30 minutes. ORO staining was detected using Texas Red excitation filter (540–580 nm) in epifluorescence.

### Statistical Methods

Values are expressed as mean ± SEM for replicates between experiments. Data were analyzed using Student’s t-tests with significance set at P<0.05. Statistical software packages used for analysis include GraphPad Prism 5 and IBM SPSS Statistics v19.

## Results

### NA Suppresses LPS-induced Transcription of Pro-inflammatory Mediators

**Figure 1:**
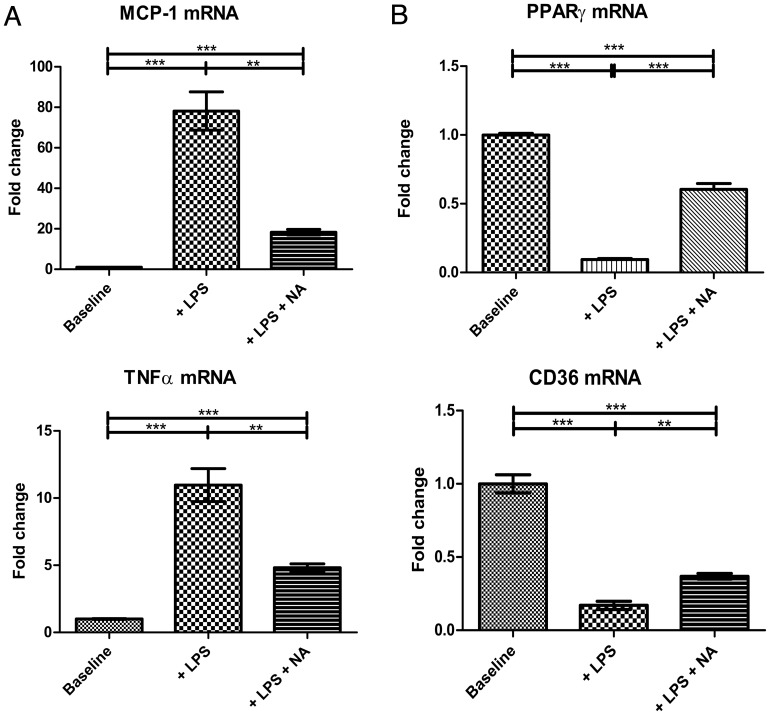
Effects of nicotinic acid (NA) on mRNA transcription of MCP-1, TNFα, PPARγ, and CD36. Basal THP-1 macrophages were pre-incubated with LPS prior to administration of NA versus vehicle controls. **1A** LPS increased mRNA transcription of MCP-1 and TNFα, while NA treatment attenuated this LPS-induced up-regulation (n = 3 per group). **1B** LPS suppressed mRNA transcription of PPARγ and its downstream effector CD36, yet treatment by NA effectively reversed it (n = 3 per group). *P<0.05, **P<0.01, ***P<0.001, ns = non significant.

We first established the response to NA in basal state (i.e. non-foam cell) macrophages. The TLR-4 agonist lipopolysaccharide (LPS) was used as an inflammatory stimulus. THP-1 macrophages were incubated with LPS (50 ng/mL) for 18 hours, followed by 1×10^−4^ M NA for 24 hours. Previous dose-response experiments (1×10^−3^ to 10^−6^ M) established optimal dosing of NA and excluded detrimental effects on cell viability [24]. Pre-incubation with LPS increased mRNA transcription of pro-inflammatory cytokines: MCP-1 by 80-fold (P<0.001, n = 3 per group); and TNFα by 10-fold (P<0.001, n = 3 per group) (Figure 1A). As we have previously reported, NA treatment significantly attenuated this LPS-induced transcriptional up-regulation by 76.6±12.2% for MCP-1 (P<0.01, n = 3 per group), and by 56.1±11.5% for TNFα (P<0.01, n = 3 per group), confirming the anti-inflammatory effect of NA on basal non-foam cell macrophages.

### NA Restores LPS-induced Transcriptional Suppression of PPARγ in Basal Macrophages

PPARγ contributes to the regulation of reverse cholesterol transport apparatus and cellular lipid metabolism. We first established that pre-incubation with LPS suppressed PPARγ and its downstream effector CD36 mRNA transcription by 90% (P<0.001, n = 3 per group) and 83% (P<0.001, n = 3 per group), respectively (Figure 1B). This LPS-induced transcriptional suppression was effectively reversed by treatment with NA, which increased PPARγ by 536.5±46.4% (P<0.001, n = 3 per group) and CD36 by 116.8±19.8% (P<0.01, n = 3 per group).

### GW1929 but not NA Promotes Cholesterol Efflux from Macrophage-derived Foam Cells

**Figure 2:**
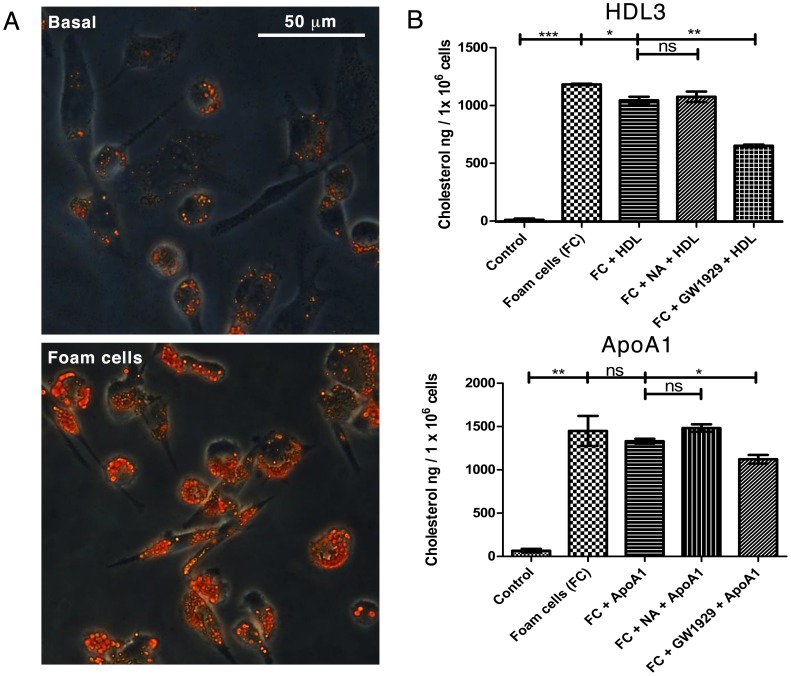
Foam cell induction and the effects of NA and GW1929 on foam cell cholesterol efflux. **2A** Phase-contrast microscopy of Oil-red-O staining of THP-1 cells in basal (upper) and foam cells (lower) states, confirming foam cell induction. **2B** Foam cells were treated with NA, GW1929, or vehicles only for 24 hours, followed by cholesterol efflux facilitated by HDL3 or ApoAI in the culture medium. Cholesterol efflux was studied by measuring cellular cholesterol content after hexane:isopropanol extraction. GW1929 treatment reduced extracted cellular cholesterol in GW1929+ HDL group versus with HDL alone; and in GW1929+ ApoAI group versus with ApoAI alone (n = 2 per group). NA treatment had no effect on extracted cellular cholesterol content at a range of NA concentration 1×10^−3^ to 10^−6^ M compared to either HDL3 or ApoAI alone groups. (Representative data at 1×10^−4^ M NA concentration shown). *P<0.05, **P<0.01, ***P<0.001, ns = non significant.

Activation of PPARγ facilitates reverse cholesterol transport (RCT) by up-regulation of ABC transporter proteins via recruitment and dimerization of another nuclear transcription factor, LXRα [28]. Since NA activated PPARγ in basal macrophages, we hypothesized that NA might promote RCT in macrophage-derived foam cells. Basal macrophages were induced into foam cells, confirmed by Oil-red-O staining in sample wells (Figure 2A). Foam cells were treated with NA, GW1929 or vehicles only for 4, 24, or 48 hours, followed by cholesterol efflux for 24 hours facilitated by cholesterol acceptor HDL3 or ApoAI in the culture medium. As anticipated, direct activation of PPARγ by GW1929 resulted in significantly higher cholesterol efflux as demonstrated by a reduction in extracted cellular cholesterol by 37.7±3.1% in GW1929+ HDL group compared to incubation with HDL alone (P<0.01, n = 2 per group); and by 15.6±4.3% in GW1929+ ApoAI group compared to incubation with ApoAI alone (P<0.05, n = 2 per group) (Figure 2B). Corresponding cholesterol contents in efflux supernatants were increased by 15.8±2.7% in GW1929+ HDL group (P<0.05, n = 2 per group); though no significant change was detected in the GW1929+ ApoAI group. Nicotinic acid treatment had no effect on either extracted cellular cholesterol or supernatant cholesterol content at a range of NA concentration 1×10^−3^ to 10^−6^ M compared to either HDL or ApoAI alone groups. (Representative data at 1×10^−4^ M NA concentration of 24 hours treatment shown in Figure 2B; no difference was detected in 3 repeat experiments, each with n = 4 per group. Data from other time points shown in Figure S2).

### Effect of NA and GW1929 on Reverse Cholesterol Transport Apparatus: LXRα, ABCA1 and ABCG1

**Figure 3:**
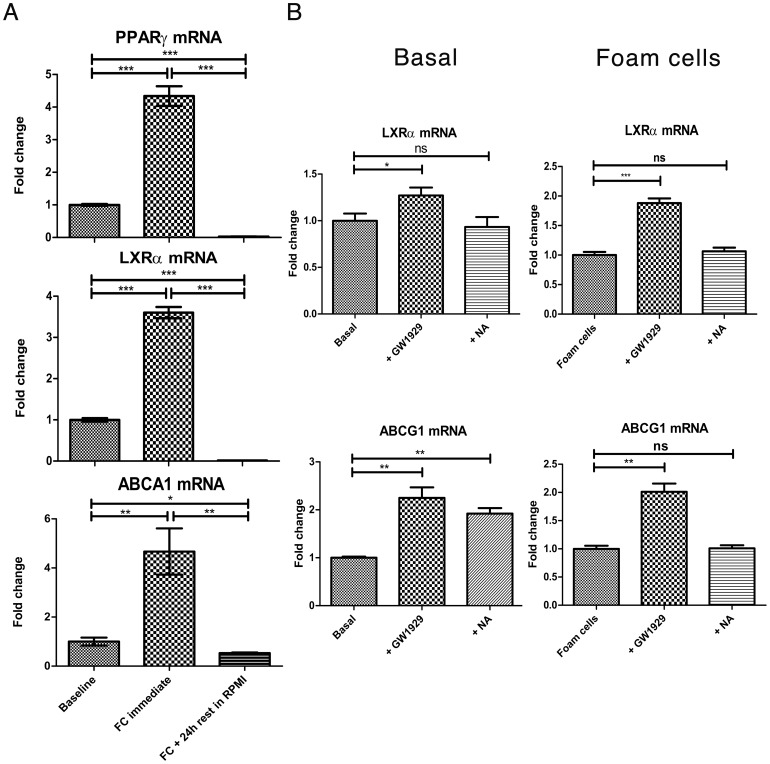
Effects of NA and GW1929 on reverse cholesterol transport apparatus: LXRα, ABCA1 and ABCG1. **3A** mRNA transcription of all three genes were significantly up-regulated immediately following cholesterol loading but returned to baseline or below-baseline level after resting in standard RPMI media for 24 hours. **3B** In basal macrophages, NA significantly up-regulated ABCG1 mRNA transcription (n = 3 per group); this effect was not seen in foam cells. PPARγ activation using GW1929 significantly up-regulated mRNA transcription with both NR1H3 and ABCG1 in both basal macrophages and foam cells. *P<0.05, **P<0.01, ***P<0.001, ns = non significant.

In order to elucidate why NA had no effect on RCT in foam cells, we investigated the effect of NA and GW1929 on genes in the PPARγ-LXRα-ABC transporters pathway. We first confirmed that PPARγ, NR1H3 (for LXRα), and ABCA1 were indeed all lipid sensitive genes using qRT-PCR. mRNA transcription of all three genes were significantly up-regulated by cholesterol loading. Immediately following 48 hours of 50 µg/mL ac-LDL incubation, PPARγ mRNA transcript was significantly increased by 4.3-fold (P<0.001); NR1H3 by 3.6-fold (P<0.001); and ABCA1 by 4.7-fold (P<0.01) (Figure 3A). However, after resting these induced foam cells in standard media (RPMI supplemented with 10% fetal bovine serum and 0.05 mM 2-mercaptoethanol) for 24 hours after induction, the mRNA transcript levels of all 3 genes returned to baseline or below-baseline levels. This time point was adopted for subsequent experiments given the intention to test the potential for gene up-regulation upon treatment. Basal macrophages and foam cells were treated with 1×10^−4^ M NA or 2×10^−6^ M GW1929 versus vehicles for 4 or 24 hours. In basal macrophages, NA significantly up-regulated ABCG1 mRNA transcription at 24 hours by 92.1±11.8% (P<0.001, n = 3 per group). In foam cells, however, NA showed no effect on transcriptional regulation (Figure 3B). This contrasts with direct PPARγ activation using GW1929, where significant transcriptional activation was seen with both NR1H3 and ABCG1 in both basal macrophages and foam cells at 4 hours and 24 hours.

### PPARγ Pathway is Activated by NA in Basal Macrophages but not in Foam Cells

**Figure 4:**
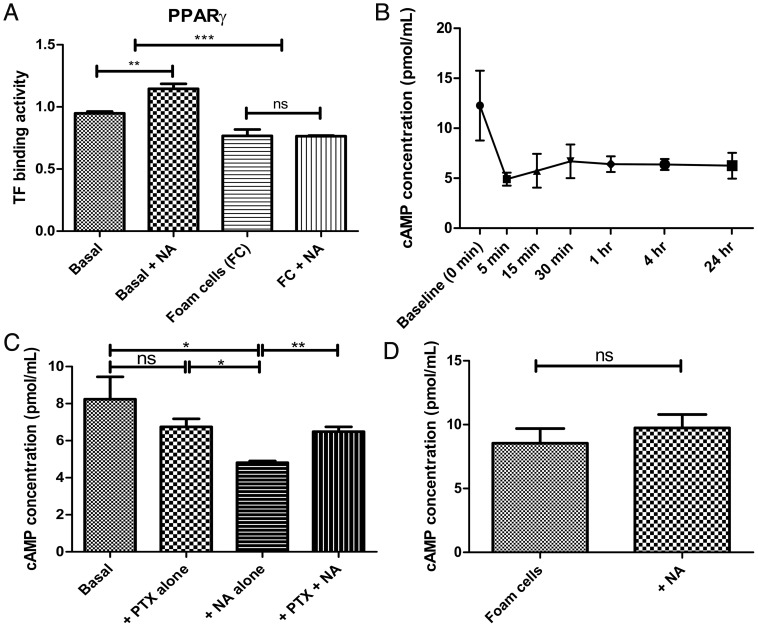
Effects of NA on PPARγ and cAMP pathways in basal versus foam cells. **4A** PPARs nuclear binding ELISA showed that cholesterol loading reduced PPARγ transcription factor binding (n = 6 per group); and that NA significantly increased PPARγ binding in basal macrophages (n = 3 per group) but not in foam cells. **4B** NA (1×10^−4^ M) rapidly reduced cAMP level and this effect persisted for 24 hours. **4C and 4D** Pre-incubation with pertussis toxin (PTX) or vehicles for 18 hours followed by NA. In basal macrophages, cAMP level diminished in NA alone group compared to control (n = 3 per group) and this effect was abolished by PTX pre-incubation. In contrast, NA did not affect cAMP level in foam cells (n = 3 per group). *P<0.05, **P<0.01, ***P<0.001, ns = non significant.

In view of the discrepancy of ABC transporters activation between GW1929 and NA treatment, we sought to explore putative cellular mechanisms by which ABCA1 and ABCG1 may be regulated by NA. Having already established that NA could reverse LPS-induced suppression of PPARγ and CD36 mRNA transcription in basal THP-1 macrophages, we tested, in the absence of LPS, direct PPARα, δ, γ transcription factor binding activity (TFBA) activated by 1×10^−4^ M NA in basal macrophages and foam cells using ELISA. PPARα TFBA was significantly increased in foam cells compared to basal macrophages by 36.3±6.2% (P<0.001, n = 6 per group), whereas PPARδ and PPARγ TFBA were both significantly diminished in foam cells compared to basal macrophages by 25.8±3.6% (P<0.001, n = 6 per group), and 26.9±5.1% (P<0.001, n = 6 per group), respectively. Interestingly, NA significantly increased PPARγ TFBA in basal macrophages by 20.8±4.5% (P<0.01, n = 3 per group) (Figure 4A). This PPARγ activation in basal non-LPS stimulated macrophages by NA was further confirmed by quantitative RT-PCR showing up-regulation of PPARγ downstream effector CD36 by 73.7±12.6% (P<0.01, n = 3 per group). In contrast, no effect on PPARγ TFBA or CD36 mRNA transcription was seen in foam cells.

### Cyclic AMP is Suppressed by NA in Basal Macrophages, but not in Foam Cells

Another potential mechanism for NA-induced activation of ABC transporters is via G-protein mediated cyclic AMP regulation [29]. NA activates GPR109A, a G_i_-protein coupled receptor, and reduces cAMP in adipocytes [30]. We found cAMP level in basal THP-1 macrophages dropped within 5 minutes after the application of 1×10^−4^ M NA and this reduction persisted for at least 24 hours (Figure 4B). We then pre-incubated basal macrophages and foam cells with 100 ng/mL pertussis toxin (PTX, to inhibit G_i/o_ coupling) or vehicle-only for 18 hours followed by 1×10^−4^ M NA for 1 hour. In basal macrophages, cAMP level was significantly diminished in NA alone group compared to control by 41.5±14.8% (P<0.05, n = 3 per group) and this effect was abolished by PTX pre-incubation, confirming the role of G_i/o_-protein signaling involved in NA activation (Figure 4C). In contrast, NA did not affect cAMP level in foam cells (Figure 4D).

### GPR109A Expression is Lost in Foam Cells *in vitro*

**Figure 5:**
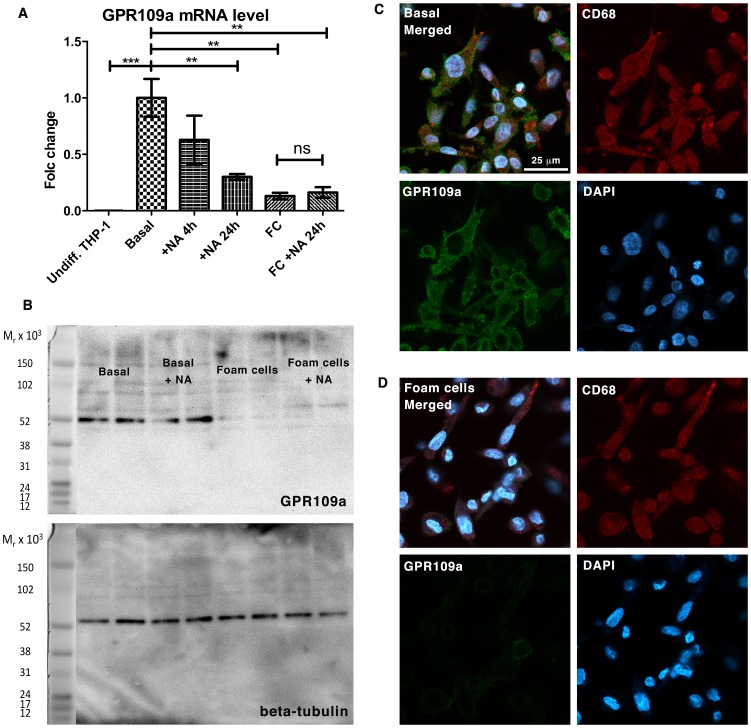
Down-regulation of GPR109A in human macrophage-derived foam cells. **5A** GPR109A mRNA was not expressed in undifferentiated THP-1 cells but expression was seen after differentiation into basal macrophages. Treatment with NA for 24 hours caused significant down-regulation of GPR109A mRNA transcription (n = 3 per group). Foam cells induction down-regulated GPR109A mRNA transcription compared to basal macrophages (n = 3 per group). **5B** Western blot showed the loss of protein expression of GPR109A in total cell lysates from foam cells compared to basal macrophages. **5C and 5D** Confocal fluorescence immunocytochemistry demonstrated the expression of GPR109A on the cell surface in cultured THP-1 basal macrophages (**5C**), and the down-regulation of GPR109A expression after foam cell induction (**5D**).

Since neither cAMP nor PPARγ pathways were influenced by NA in foam cells, we examined the expression pattern of GPR109A in cell culture. Using quantitative real-time RT-PCR, we showed that GPR109A mRNA was not expressed in undifferentiated THP-1 cells but expression was seen after differentiation into basal macrophages (Figure 5A). Treatment with NA for 24 hours, as expected with G-protein coupled signaling, caused desensitization and significant down-regulation of GPR109A mRNA transcription by 70±16.9% (P<0.01, n = 3 per group). Surprisingly, foam cell induction alone caused an even more dramatic down-regulation of GPR109A mRNA transcription by 87±17% compared to basal macrophages (P<0.01, n = 3 per group). To confirm this finding at a protein level, we performed Western blot and fluorescence immunocytochemistry on basal macrophages and foam cells. Figure 5B showed the loss of protein expression of GPR109A in total cell lysates from foam cells compared to basal macrophages in a Western blot; whereas Figure 5C and 5D demonstrated the expression of GPR109A on the cell surface in cultured THP-1 basal macrophages (5C), and the down-regulation of GPR109A expression after foam cell induction (5D).

### GPR109A is Expressed in Human Carotid Plaques but its Expression is Down-Regulated in Carotid Plaque Foam Cells

**Figure 6:**
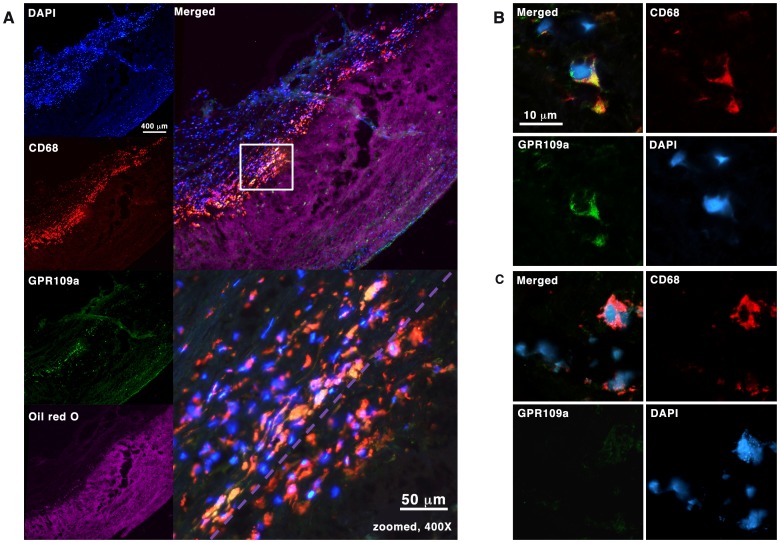
Expression of GPR109A in ex-vivo human carotid plaques. **6A** Fluorescence immunohistochemistry staining with antibodies against CD68 and GPR109A. Immediately adjacent 10 µm cryosection was stained with Oil Red O for lipid distribution and visualized using Texas Red excitation filter (540–580 nm) in epifluorescence. CD68-positive cells were seen clustering at the interface between lipid-rich region and the overlying fibrous cap. GPR109A co-expression was seen in a sub-population of these CD68-positive cells outside of the lipid-rich region (yellow). Purple dotted line represents the boundary of the lipid-rich region as seen in the image above. **6B and 6C** Loss of GPR109A expression in lipid-rich region. Confocal fluorescence images of CD68-positive cells outside of lipid-rich region are shown in **6B**; and those within the lipid-rich region, likely to represent foam cells, are shown in **6C**.

**Figure 7:**
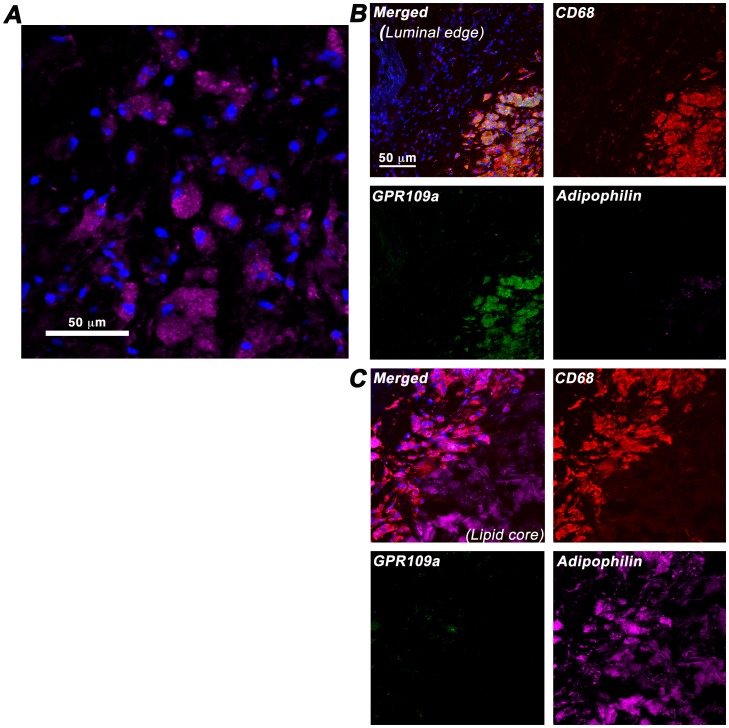
Down-regulation of GPR109A in ex-vivo lipid-laiden plaque foam cells. Adipophilin (perilipin 2) staining is specific to intra-cellular lipid-droplet associated protein and represents a sensitive marker of foam cells. **7A** High-powered confocal image showed the outline of intra-cellular lipid droplets. **7B** GPR109A is strongly co-expressed in CD68-positive plaque macrophages without extensive lipid-loading i.e. non-foam cells; whereas plaque foam cells, which showed strong co-expression of CD68 and adipophilin due to extensive lipid-loading as shown in **7C**, do not co-express GPR109A.

Finally, to ascertain whether these *in-vitro* findings are of potential clinical significance, we analysed human carotid atherosclerotic plaques from endarterectomy. Five recently symptomatic carotid plaques were obtained for histological and fluorescence immunohistochemistry. At the region of maximal luminal stenosis, 10 µm cryosections were double-stained with antibodies against CD68 and GPR109A; with the immediately adjacent section stained with Oil-red-O to visualise plaque lipid distribution. As shown in Figure 6A, CD68-positive cells were consistently observed clustering at the interface between the lipid-rich area and the overlying fibrous cap layer; and a sub-population of these CD68-positive cells also expressed GPR109A. More importantly, GPR109A co-expression was observed in CD68-positive cells outside of the lipid-rich area, whereas CD68-positive cells within the lipid-rich area, which are highly likely to represent macrophage-derived foam cells, do not co-express GPR109A. Figure 6B and 6C showed confocal images of the co-expression of GPR109A and CD68 in basal macrophages outside of lipid core (6B), and the markedly diminished GPR109A co-expression in CD68-positive macrophages inside the lipid-rich area (6C). To further confirm our finding, we performed co-localisation experiment using antibodies against CD68, GPR109A, and a lipid-droplets associated protein adipophilin to identify foam cells. Figure 7B showed that GPR109A-positive cells have low content for lipid droplets i.e. are non-foam cell macrophages; whereas Figure 7C demonstrated CD68-positive cells with high lipid-droplets content i.e. “foam cells” macrophages do not co-express GPR109A.

## Discussion

Nicotinic acid drives atherosclerosis regression in humans and acts on monocytes via GPR109A to up-regulate mediators of cholesterol efflux. Recent observations in mice suggest that atherosclerosis progression can be attenuated by nicotinic acid independently of any effect on lipoproteins [25]. These observations suggested that nicotinic acid may exert anti-atherosclerotic effects by directly promoting cholesterol efflux from foam cells. Here, we tested that hypothesis and demonstrated that, while PPARγ agonism enhanced cholesterol efflux, NA failed to do so due to down-regulation of the receptor GPR109A in foam cells as compared with basal macrophages. Using samples obtained at carotid endarterectomy, we demonstrated that a similar phenomenon occurs *in vivo*. Our findings imply that direct NA-promotion of cholesterol efflux from foam cells does not contribute to atherosclerosis regression that has been documented with this drug. The patho-physiological relevance of GPR109A expression in monocytes/macrophages and its subsequent down-regulation in foam cells in the context of plaque regression remain to be established.

We studied the effect of NA on peroxisome proliferator-activated receptors (PPARs), which are members of a nuclear receptor protein family that control transcription cascades in both glucose and lipid metabolism. Both PPARα and PPARγ are implicated in the regulation of cellular lipid metabolism [31], with PPARγ-LXRα-ABC transporters pathway being the most studied [28]. Consistent with previous *in-vitro* studies in non-foam cell macrophage/monocytic cell lines [16], [17], we showed that NA has a stimulatory effect on PPARγ expression and nuclear activation with enhanced transcription of one of its downstream effector, CD36; and of the cellular cholesterol efflux ABC transporters. As observed recently in mice by Lukasova *et al.* [25], we did not detect enhanced transcription with ABCA1 with NA, but ABCG1 transcription was significantly up-regulated by NA in basal macrophages. However, we showed that NA loses this stimulatory effect on the RCT apparatus as a result of GPR109A receptor down-regulation after foam cell induction, as well as being unable to affect functionally cellular cholesterol efflux in cultured macrophage-derived foam cells.

NA also suppressed cAMP in non-stimulated, basal monocytic cells, through a mechanism that was pertussis toxin-sensitive, which deactivates G_i_ and G_o_ G-proteins. Although this is contrary to Rubic *et al* [16], who showed a rise in cAMP associated with enhanced cholesterol efflux from basal macrophages by NA, our results are in agreement with several other groups who have observed NA-mediated inhibition of the cAMP response [32], [33] and similar effects are also seen in HM74a-transfected CHO-K1 [30], 293EBNA [34], HEK293 and 3T3L1 adipocytes [35], which are at least partially ascribed to inhibition of adenylate cyclase. We demonstrated that this classic cAMP response observed in basal monocytic cells is absent in transformed macrophage-derived foam cells.

Our results showed, for the first time, the expression of GPR109A in a subpopulation of CD68-positive plaque macrophages in human carotid plaques. Moreover, these GPR109A and CD68 co-expressing cells appear to cluster just outside of lipid-rich region in plaques at the interface between the lipid pool and the overlying fibrous cap. Our findings are supported by a recent animal study in LDLR^−/−^ mice, which reported co-expression of a red fluorescent protein (RFP) transgene placed under GPR109A promoter control and a macrophage marker MOMA-2 in murine aortic plaques [25]. These findings provide a plausible target in human plaques for the direct GPR109A-mediated anti-atherosclerotic effects of NA described recently [24] [25]. Adipophilin (perilipin 2) has recently been established as a sensitive marker of monocyte lipid loading [36], [37] and that its protein expression is increased in symptomatic carotid atherosclerosis [38]. Using immunofluorescence against adipophilin, we further confirmed that GPR109A expression in symptomatic human carotid plaques is down-regulated in CD68-positive cells that are laden with intra-cellular lipid-droplets, characteristic of foam cells.

There are limitations in our study, which we have attempted to address. In particular, GPR109A and GPR109B receptors are highly homologous and are 95% identical on the amino acid level [39]. The detection of specific GPR109A expression in Western blot and on immunohistochemistry is thus challenging. However, we have recently demonstrated that the anti-GPR109A antibody used in these experiments preferentially targets GPR109A such that its binding on a Western blot was substantially attenuated when GPR109A was specifically knocked down using siRNA [24]. In addition, although GPR109A and GPR109B share high degree of homology, only GPR109A binds nicotinic acid with high affinity. In our cholesterol efflux study, we chose not to evaluate free cholesterol and esterified cholesterol (EC) separately, since early study has estimated that up to 30% of the cellular EC pool in THP-1 foam cells was derived directly from un-degraded AcLDL-derived EC, hence bypassing the normal hydrolysis/re-esterification cycle [40]. Therefore, attempts to separately evaluate free cholesterol and EC would be confounded and would make a valid interpretation difficult. In addition, one interesting observation from our RCT gene expression experiment also remained unexplained. After the initial up-regulation following Ac-LDL incubation, both PPARγ and its heterodimerisation partner LXRα mRNA transcription appeared to be down-regulated (beyond baseline) in the resting phase (Figure 3A). Although we found no clear explanation from the literature, we were reassured by our subsequent complete PPARα, γ, δ transcription factor binding ELISA assay, which also demonstrated a down-regulation of PPARγ transcription factor activity following the same foam cell induction protocol (Figure 4A). Peculiarly, PPARα transcription factor activity was enhanced by the same foam cell treatment. Therefore, although the PPAR regulatory mechanisms remain illusive, our foam cell induction protocol, with its resting phase, produced viable foam cells that displayed diverse PPAR responses. Moreover, stimulation of induced foam cells with GW1929 elicited the anticipated effect of LXRα and ABCG1 mRNA transcription up-regulation (Figure 3B, right column), further supporting that the induced foam cells behaved appropriately. The transcriptional regulation of nuclear factor PPARα, γ, and δ is complex [41], [42] and beyond of scope of our current study. The authors would like to postulate that Ac-LDL, being itself a pro-inflammatory stimulus, may stimulate the release of pro-inflammatory cytokine such as TNF-α and IL-1β, which is known to down-regulate PPARγ mRNA transcription [43], [44]. During the resting phase, when the overwhelming lipid loading stimulus was withdrawn, the pro-inflammatory after-effect might have contributed to the “over-shot” phenomenon as seen with PPARγ mRNA transcription and that of its partner LXRα.

Besides its potential pharmacological value, there remain important biological questions as to the physiological role of GPR109A, for which the cognate ligand is believed to be β-hydroxybutyrate [45]. β-hydroxybutyrate is a ketone body that is produced from acetyl-CoA in hepatocytes and is an alternative energy source to the brain and, to a lesser extent, the heart when glucose availability is low during fasting or starvation. Activation of GPR109A in adipocytes inhibits fatty acid release, possibly acting as a negative feedback against excessive lipolysis in starvation [46]. Indeed plasma concentration of ketones can change over several orders of magnitude under normal physiology [21]. However, the physiological function of GPR109A in immune cells is still largely unknown. Activation of GPR109A by the bacterial fermentation product, butyrate, has been shown to exert tumour-suppressing effects in colon cancer [47]; while in cerebral hypoxia, β-hydroxybutyrate is shown to be neuroprotective [48]–[50]. Although this may reflect adaptations to metabolic substrate utilisation/energetics, immune cell modulation via GPR109A is another plausible explanation.

The co-expression of GPR109A in CD68-positive immune cells in human plaques at the interface between lipid pool and the overlying fibrous cap may suggest a regulatory cross-talk between ‘metabolic’ and ‘inflammatory’ pathways, which is increasingly appreciated [51]. We have previously shown anti-inflammatory effects of NA in both adipocytes [23] and stimulated monocytes [24], those being the two principal cell types bearing GPR109A. Each of these cell types is implicated in inflammatory pathologies relating to obesity and/or high caloric intake. It is therefore plausible that a metabolic mediator that is regulated and released by the liver over a large dynamic range in starvation might be involved in the suppression of inflammation in these cell types. Although no direct measurement of β-hydroxybutyrate has been made in atherosclerotic plaque, the selective expression of GPR109A in macrophages suggests that it, or an alternative ligand, may have a role in immunomodulation in that context.

Finally, it is important to allude to the potential mechanisms of GPR109A down-regulation in foam cells. As with most G-protein coupled signaling, GPR109A is negatively regulated upon receptor stimulation. Ligand binding of GPR109A causes activation of G-protein coupled receptor kinases (GRKs), which in turn phosphorylate the occupied receptor [52]. The phosphorylated GPR109A is then susceptible to binding of regulatory adaptor protein β-arrestins. In addition to direct attenuation of GPCR-signalling (desensitization), binding of β-arrestins also recruits AP2 and clathrin to facilitate endocytosis via clathrin-coated pits and recycling of desensitized GPCR [53]. Moreover, it has recently been shown that β-arrestins contribute to the PGD_2_-mediated cutaneous flushing associated with NA [54], and antagonize NF-κB signaling [55], both independent of the classic G-protein signaling. As a result, although little is currently known with regards to β-arrestin signaling in foam cells, its involvement in G-protein coupled receptor down-regulation, and its direct involvement in downstream cell signaling, make β-arrestins an attractive target for future investigation.

### Conclusions

In conclusion, this study shows that despite its potent anti-inflammatory effect on human macrophages, nicotinic acid has no direct effect on reverse cholesterol transport in macrophage-derived foam cells; and this may be explained by the down-regulation of its receptor GPR109A upon foam cell transformation. This implies that direct NA-promotion of cholesterol efflux from foam cells does not contribute to atherosclerosis regression, which has been demonstrated with this drug. Given the cellular distribution of GPR109A/CD68 co-expressing macrophages in human carotid plaques, the patho-physiological significance of GPR109A expression in human plaque macrophages and its down regulation in foam cells remains to be elucidated.

## Supporting Information

Figure S1 **Diagrammatic representation of cell treatment in cholesterol loading and efflux experiments.** All treatment groups had identical exposure to PMA and identical culture duration. Cell treatments were identical in qRT-PCR, cholesterol efflux, Western blot (WB), and immunofluorescence (IF) staining experiments; in qRT-PCR experiments, treatment with NA and GW1929 were applied at the end (at asterisk *) in line with foam cells (FC) group to ensure robust comparison.
(TIF)

Figure S2 **Effect of NA on foam cells at different concentration and at different time points.** Cholesterol efflux was studied by measurement extracted cholesterol content after hexane:isopropanol extraction. Nicotinic acid (NA) has no effect on cholesterol efflux compared to using HDL alone at 4 hours (**S2A**) and at 48 hours (**S2B**). Data at 24 hours is shown in main Figure 2. NA concentration: NA-3 = 1×10^−3^ M, NA-4 = 1×10^−4^ M, NA-5 = 1×10^−5-^ M, NA-6 = 1×10^−6^ M. Time indicated duration of NA treatment.
(TIF)
