# Supplementary figures and images for: Nicotinic Acid Receptor GPR109A Is Down-Regulated in Human Macrophage-Derived Foam Cells
Source: PLoS One. 2013 May 2;8(5):e62934. doi: 10.1371/journal.pone.0062934 (PMC3642175; doi:10.1371/journal.pone.0062934)

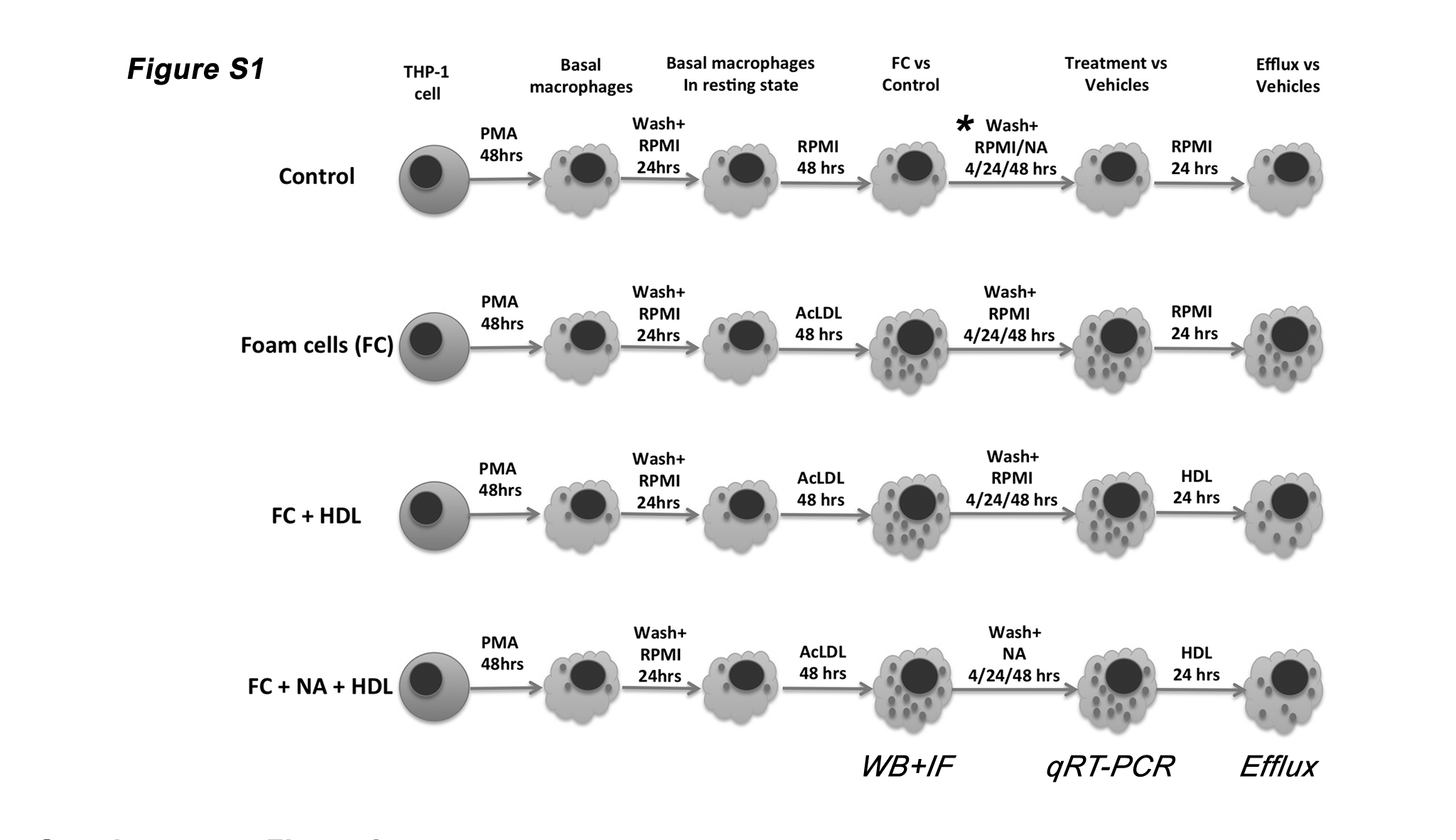

Supplement: Figure S1 — Diagrammatic representation of cell treatment in cholesterol loading and efflux experiments. All treatment groups had identical exposure to PMA and identical culture duration. Cell treatments were identical in qRT-PCR, cholesterol efflux, Western blot (WB), and immunofluorescence (IF) staining experiments; in qRT-PCR experiments, treatment with NA and GW1929 were applied at the end (at asterisk *) in line with foam cells (FC) group to ensure robust comparison. (TIF) [file pone.0062934.s001.tif]

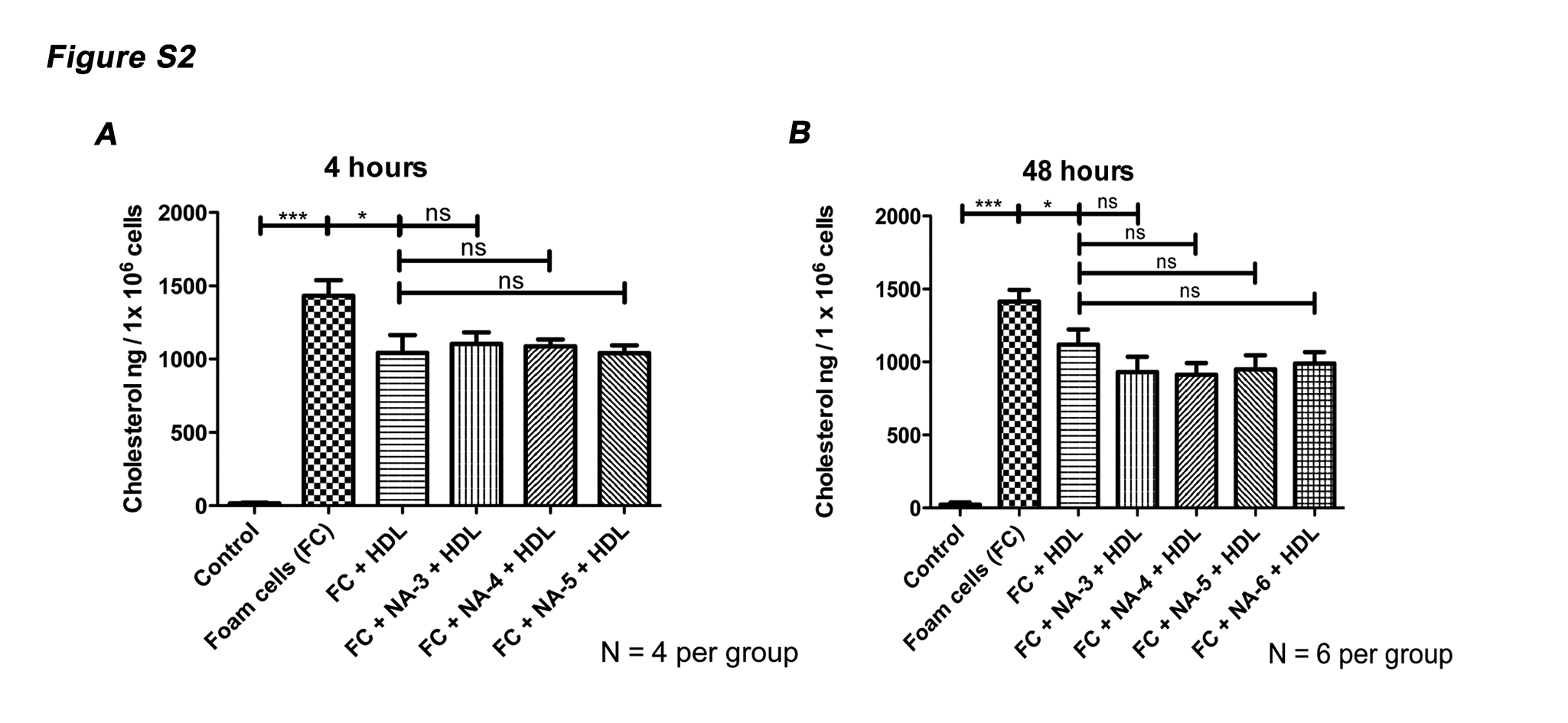

Supplement: Figure S2 — Effect of NA on foam cells at different concentration and at different time points. Cholesterol efflux was studied by measurement extracted cholesterol content after hexane:isopropanol extraction. Nicotinic acid (NA) has no effect on cholesterol efflux compared to using HDL alone at 4 hours (S2A) and at 48 hours (S2B). Data at 24 hours is shown in main Figure 2. NA concentration: NA-3 = 1×10^−3^ M, NA-4 = 1×10^−4^ M, NA-5 = 1×10^−5^- M, NA-6 = 1×10^−6^ M. Time indicated duration of NA treatment. (TIF) [file pone.0062934.s002.tif]
